# Effects of different fatty acids composition of phosphatidylcholine on brain function of dementia mice induced by scopolamine

**DOI:** 10.1186/s12944-016-0305-5

**Published:** 2016-08-24

**Authors:** Miao-miao Zhou, Yong Xue, Shu-hong Sun, Min Wen, Zhao-jie Li, Jie Xu, Jing-feng Wang, Teruyoshi Yanagita, Yu-ming Wang, Chang-hu Xue

**Affiliations:** 1 College of Food Science and Engineering, Ocean University of China, NO. 5 Yushan Road, Qingdao, Shangdong Province 266003 People’s Republic of China; 2 Clinical Laboratory, Qingdao Municipal Hospital (Group), NO. 1 Jiaozhou Road, Qingdao, Shangdong Province 266011 People’s Republic of China; 3 Department of Health and Nutrition Sciences, Faculty of Health and Social Welfare Sciences, Nishikyushu University, 4490-9 Ozaki, Kanzaki, Saga 842-8585 Japan

**Keywords:** Phosphatidylcholine, Cholinergic system, Long-chain n-3 polyunsaturated fatty acids, Antioxidant

## Abstract

**Background:**

Phosphatidylcholine (PC), the major source of dietary choline, has been demonstrated to improve the capability of learning and memory in rodent and the amelioration of long-chain n-3 polyunsaturated fatty acids (PUFA) on anti-aging and anti-oxidation is widely known as well. In this study, three kinds of PC were chose to demonstrate the role of different fatty acids composition on glycerol backbone in improving the brain function of mice induced by scopolamine which was used to impair cholinergic system and cause oxidative stress.

**Methods:**

Male BALB/c mice were randomly divided into 5 groups: model (M) group, control (Con) group, egg yolk lecithin (EL) group, squid PC (SQ-PC) group and sea cucumber PC (SC-PC) group. The intraperitoneal injection of scopolamine hydrobromide (5 mg/kg) was carried out on the 8^th^ of group feeding and sustained daily until the end of test. Morris water maze test was used to evaluate the improvement of cognitive decline and the activity of acetylcholinesterase (AchE), superoxide dismutase (SOD) and monoamine oxidase (MAO) and malondialdehyde (MDA) content in brain were measured to assess the physiological changes.

**Results:**

In behavior test, the latency of PC groups was significantly reduced, while number of crossing the platform and time in target quadrant were increased in comparison with M group and the improvements of SQ-PC and SC-PC were better than that of EL (*P* < 0.05). Similar trend was observed in physiological changes. The AchE activity was effectively decreased and the SOD activity increased in hippocampus, cortex and white matter when comparing PC groups with M group. SQ-PC, SC-PC and EL respectively showed 22.82, 28.80 and 11.81 % decrease in MDA level in brain compared with M group. The MAO activity in white matter of SQ-PC, SC-PC and EL group separately depressed 33.05, 33.64 and 19.73 % in comparison with M group. No significance between SQ-PC and SC-PC was found in these indicators except the SOD activity in hippocampus and white matter. SQ-PC group had a higher SOD activity in hippocampus (103.68U/mg · prot.) and lower in white matter (120.57 U/mg · prot.) than SC-PC group (95.53 U/mg · prot. in hippocampus, 134.49 U/mg · prot. in white matter). PC rich in n-3 PUFA acted more ameliorative effects than that barely contained on the indicators above.

**Conclusions:**

Different fatty acids composition of PC all could diminish the cognitive decline and biological damage and protect the brain. EPA and DHA partly enhaced to the advantageous effects.

## Background

Alzheimer’s disease (AD) is characterized by the progressive decline in memory and cognitive function [1]. It is also one of the most important forms of dementia and has become into an enormous financial burden on society with the rapid growth of elderly population in the worldwide [2]. The most disappointing and hopeless thing is that we have not found an effective treatment to cure or just suspend the disease. AD causes substantial changes in brain including synaptic and neuronal loss, Aβ deposition, hyperphosphorylation of Tau and neurobrillary tangles [3, 4]. There are many hypothesis reported about the pathogenesis of AD, in which oxidative stress and cholinergic damage are highly acceptable [5, 6]. It is a critical element that the level of brain acetylcholine (Ach) was persistently decreasing in the process of AD. The drugs that increase the content of the neurotransmitter Ach by inhibiting acetylcholine esterase for treating the AD patients are approved by U.S. Food and Drug Administration (FDA) currently [7]. However, most of these drugs should be used for a long-term condition on account of their transiently curative effect, which resulted in a series of adverse effects and suffering such as headache, insomnia and emesis [8]. The developments of preventing and curing AD through diet therapy are pregnant and necessary. It is a very appropriate way to enhance nutraceuticals in daily diet. It provides plenty of medical or health benefits such as improving the metabolism of glucose and lipid [9], changing the lipid composition of brain [10], antioxidation and anti-aging [11]. Escalating reports give us a common sense that diet supplementation with phospholipids (especially functional type) plays a positive role in protecting the nervous system and improving memory [12–14]. Meanwhile, the effect of long-chain n-3 polyunsaturated fatty acids (PUFA) of aquatic product on anti-oxidative damage is generally recognized [15]. Docosahexaenoic acid (DHA) and eicosapentaenoic acid (EPA), the main PUFA in fish oil, make the greatest contribution to the biological activities such as promoting the growth of cerebral cells and improving memory [16]. DHA is the main composition of member lipid in the nerve cells and the priority resource of fatty acid in brain cells. As an effective anti-inflammatory bioactivator, EPA has been increasingly reported to own the capability of preventing the inflammation in neurodegenerative disease [17, 18]. It is widely acknowledged that egg yolk lecithin has a good effect on mopping up free radicals and anti-aging [19]. Meanwhile phosphatidylcholine (PC) is the major source of dietary choline and it has been demonstrated that intake of PC can improve the capability of learning and memory in rats to some extent [20]. As the main component of lipid bilayer structure of cell membrane, PC acts a vital part in membrane fusion and transport [21], endocytosis and the catalytic activity of enzymes [22] and the function of lowering blood lipid and cholesterol levels, preventing cardiovascular disease and inhibiting obesity have been illuminated as well [10]. However, it is rarely illustrated whether the difference of fatty acids composition on glycerol backbone of PC molecule has influence on the improvements.

As a muscarinic anti-cholinergic drug, Scopolamine (SCO) can block the combination of neural acetyl choline (Ach) and its receptor, disrupt the transmission of cholinergic signal and lead to clinical manifestations of dementia [7]. Simultaneously, it is able to decrease the anti-oxidative enzyme activity and increase the level of radical [23]. Therefore, it becomes a widely used drug to induce dementia models by impairing short-term learning and memory [24]. SCO induces cognitive impairment with an attenuation of cholinergic neurotransmission and increase of oxidative stress [25]. Thus, nutraceuticals which can significantly improve cholinergic system and/or oxidative stress might reverse this damage. In this study, we chose three kinds of PC: egg yolk lecithin (coventional type PC), sea cucumber PC (rich in EPA) and squid PC (rich in DHA) to investigate the effect of different fatty acids composition of PC on the brain function of dementia BALB/c mice induced by intraperitoneal injection of scopolamine hydrobromide in 2-week short term test.

## Result

### Morris water maze test

**Fig. 1:**
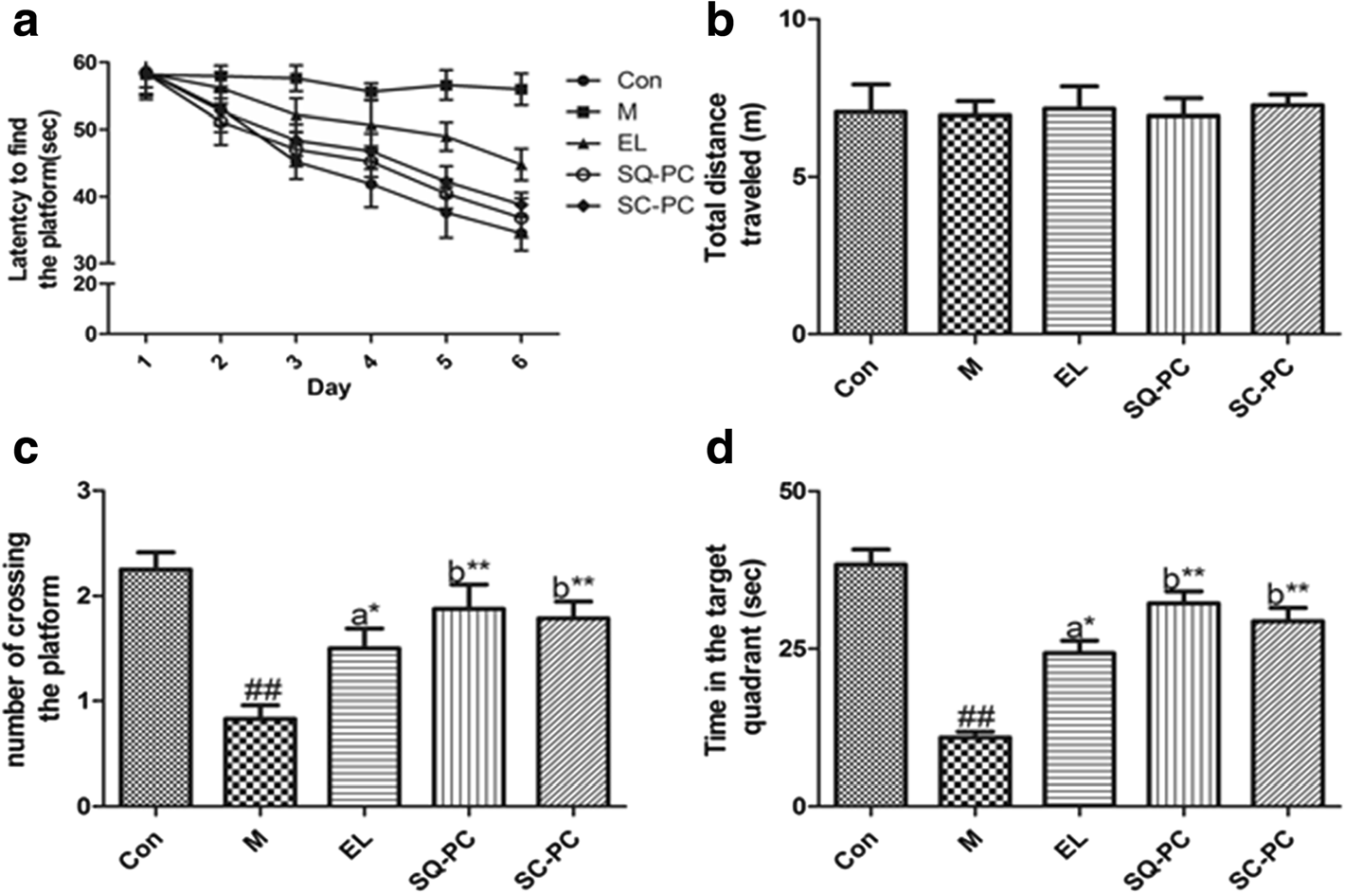
Effects of different fatty acids composition of PC on behavior test. Comparison of latency to find the platform (**a**), total distance traveled (**b**), number of crossing the platform (**c**) and time in the target quadrant (**d**) among mice with experimental diets. Results are expressed as mean ± SEM; *n* = 8 mice per group. ##*P <* 0.01, significant difference compared with Con group, *P < 0.05, **P < 0.01, significant difference compared with M group, determined by student’s *t* test. Different letters represent significant difference at *P <* 0.05 among PC groups determined by ANOVA (Tukey’s test)

As the intraperitoneal injection of Scopolamine hydrobromide was daily kept during the entire test, the time used to find the platform was becoming significant longer in M group than that in Con group during the 6-day’s training (Fig. 1). Compared to the Model group, the latency of SQ-PC group and SC-PC group mice was continuous and notablely decreased and the significance of EL group emerged on the 4^th^ (*P <* 0.05), 5^th^ (*P <* 0.01) and 6^th^ (*P <* 0.01) day of the test. In the meantime, we found a significance arose between EL group and SC-PC group as well as that between EL group and SQ-PC group and was persistent until the end of the test. Nonetheless, the difference between SQ-PC group and SC-PC group was not distinct (Fig. 1a).

No significance was found in total distance traveled of all groups mice, however the number of crossing the platform and the time in target quadrant (quadrant I) had a remarkable difference after the platform was removed from the pool (Fig. 1b). Numbers of M (*P <* 0.01) group mice crossing the platform was significantly less than that of the Con group and SQ-PC group (*P <* 0.01) and SC-PC (*P <* 0.01) group mice showed a great improvements and EL group (*P <* 0.05) also achieved partially better when compared with M group. The result of the contrast among three PC groups showed the ameliorative effect of SQ-PC group and SC-PC group was better than EL group (Fig. 1c). According to the comparison between Con group and M group mice, time in the quadrant I notly decreased from 38.39 s to 10.89 s.SQ-PC group and SC-PC group mice remained in quadrant I for respectively 32.24 s and 29.40s which meant obvious increase in contrast to M group, meanwhile EL group mice improved the time to 24.30s and also showed significance. In consistent with the performance of number of crossing the platform, SQ-PC group and SC-PC group had also achieved a better result than EL group in the time in quadrant I (Fig. 1d).

### AchE activity

**Fig. 2:**
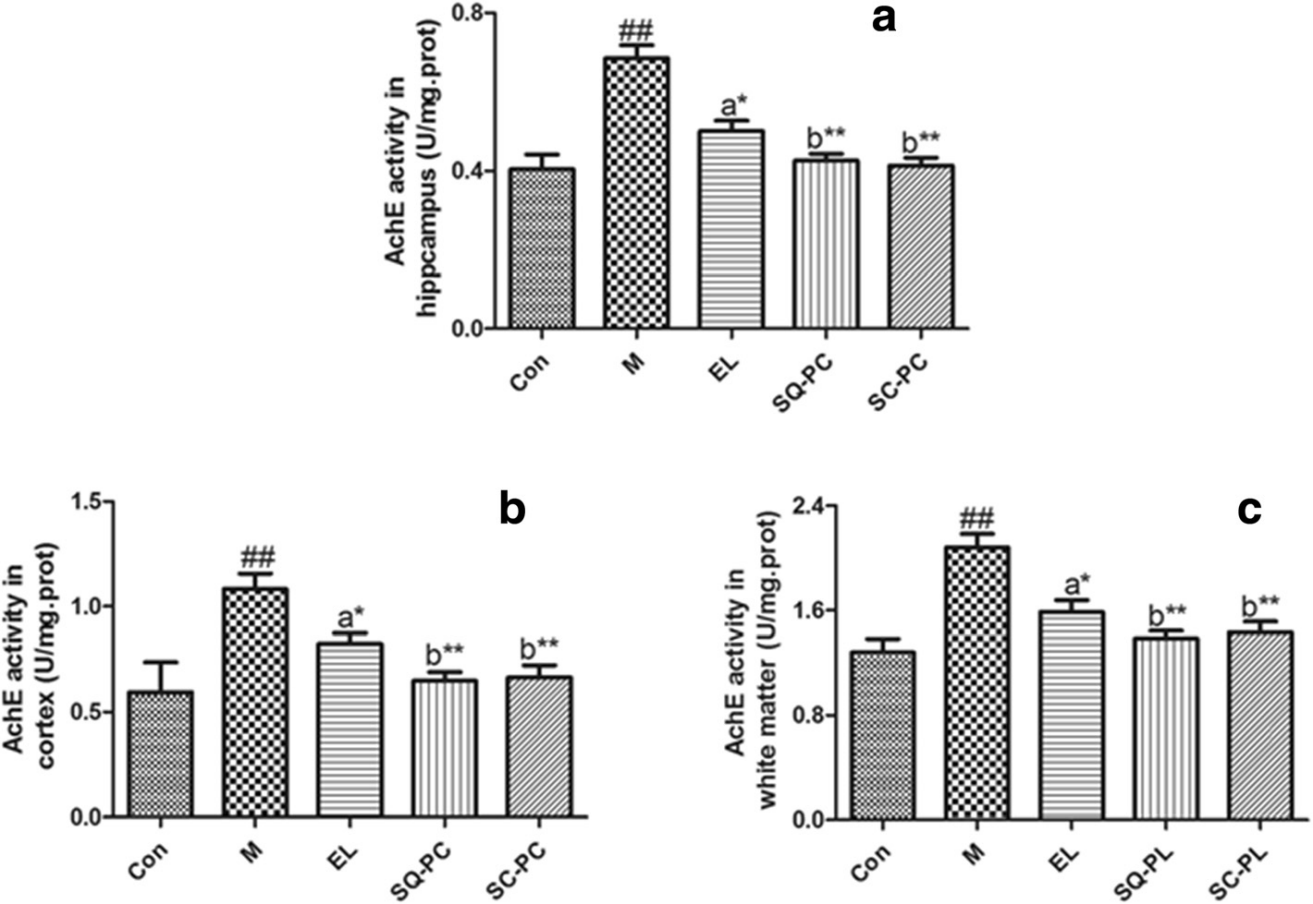
Effects of different fatty acids composition of PC on AchE activity. Comparison of AchE activity in hippocampus (**a**), cortex (**b**) and white matter (**c**) among mice with experimental diets. Results are expressed as mean ± SEM; *n* = 8 mice per group. ##*P <* 0.01, significant difference compared with Con group, *P < 0.05, **P < 0.01, significant difference compared with M group, determined by student’s *t* test. Different letters represent significant difference at *P <* 0.05 among PC groups determined by ANOVA (Tukey’s test)

Since acetylcholin esterase was one of the most relevant enzyme to acetyl choline (Ach), which was the important neurotransmitter in central cholinergic system and played a vital role in learning and memory, we investigated the AchE activity in hippocampus (Fig. 2a), cortex (Fig. 2b) and white matter (Fig. 2c). Generally, SCO-administration severely increased the AchE activity of M group (*P* < 0.01) in the areas of mice brain when compared to Con group. Contrasting the PC groups with M group, we could conclude that the PC groups all decreased the AchE activity effectively and SQ-PC group (*P* < 0.01) and SC-PC group (*P* < 0.01) showed more significant than EL group (*P* < 0.05). Similar trend of difference was observed in hippocampus, cortex and white matter with the comparison among the PC groups, that SQ-PC group and SC-PC group had a remarkable effect on decreasing the AchE activity by contrast with EL group and no significance was presented when comparing SQ-PC group and SC-PC group.

### Oxidative stress in brain

**Fig. 3:**
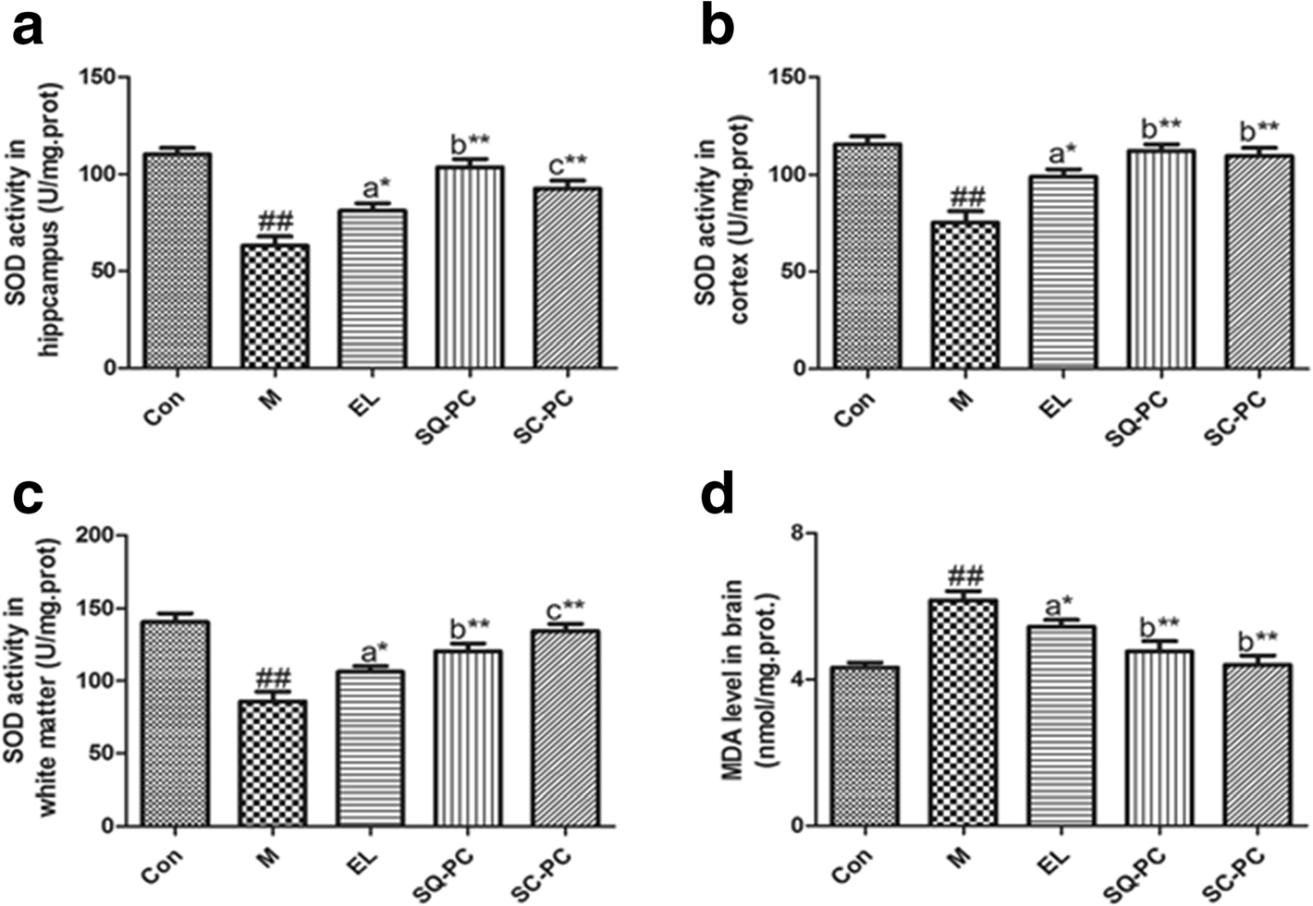
Effects of different fatty acids composition of PC on oxidative stress. Comparison of SOD activity in hippocampus (**a**), cortex (**b**) and white matter (**c**) among mice with experimental diets. **d** Comparison of MDA level in brain among mice with experimental diets. Results are expressed as mean ± SEM; *n* = 8 mice per group. ##*P <* 0.01, significant difference compared with Con group, *P < 0.05, **P < 0.01, significant difference compared with M group, determined by student’s *t* test. Different letters represent significant difference at *P <* 0.05 among PC groups determined by ANOVA (Tukey’s test)

Superoxide dismutase (SOD) activity and malondialdehyde (MDA) level, two representative oxidative stress indicators, were measured. The results showed that the administration of Scopolamine hydrobromide significantly increased the oxidative stress in M group mice as the SOD activity in cortex (*P <* 0.01), hippocampus (*P <* 0.01) and white matter (*P <* 0.01) of M group mice were markedly lower and the MDA level in brain (*P <* 0.01) was higher in comparison with Con group (Fig. 3). Compared to M group, the three kinds of PC effectively improved the SOD activity in hippocampus (Fig. 3a), cortex (Fig. 3b) and white matter (Fig. 3c).

Similar results were presented in the comparison of the effect on decreasing the MDA level (Fig. 3d) in mice brain between the PC groups and M group. Furthermore, SQ-PC group and SC-PC group showed more effective results than EL group while comparing with the PC groups. The difference between SQ-PC group and SC-PC group was not as same as that in the test above. SQ-PC group mice had a higher SOD activity in hippocampus and lower in white matter than SC-PC group. However, no significance was showed in SOD activity in cortex and MDA level in brain between the two groups.

### MAO activity

**Fig. 4:**
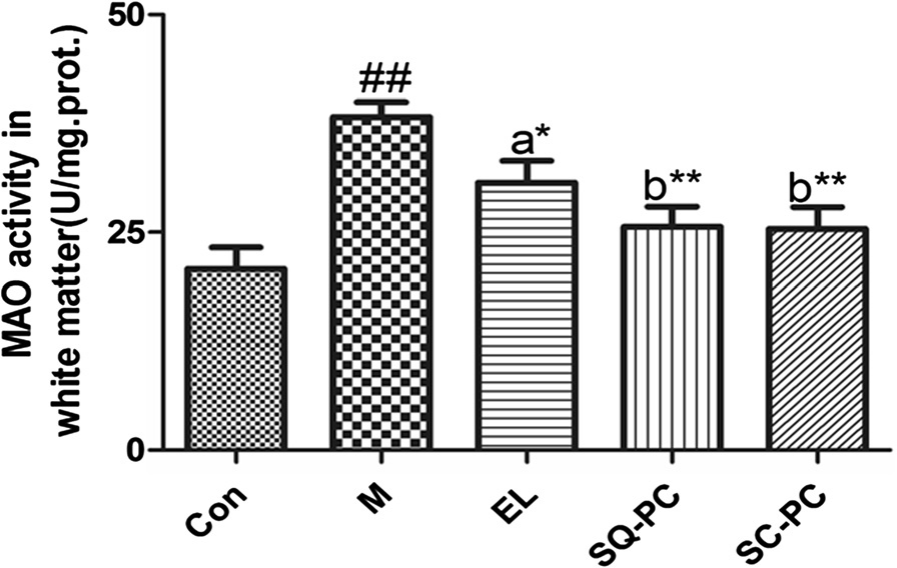
Comparison of MAO activity in white matter. Results are expressed as mean ± SEM; *n* = 8 mice per group. ##*P <* 0.01, significant difference compared with Con group, *P < 0.05, **P < 0.01, significant difference compared with M group, determined by student’s *t* test. Different letters represent significant difference at *P <* 0.05 among PC groups determined by ANOVA (Tukey’s test)

The result of MAO activity in white matter was shown in Fig. 4. M group displayed a significant increase of MAO activity in white matter when compared with Con group. EL group, SQ-PC group and SC-PC group decreased the MAO activity by 19.7 % (*P <* 0.05), 34.0 % (*P <* 0.01) and 34.6 % (*P <* 0.01) respectively in contrast to M group. No significance was found between SQ-PC group and SC-PC group and each of the two groups performed better than EL group in improving the MAO activity in white matter with the comparison between the PC groups.

## Discussion

Recently, increasing interest in improving the clinical symptoms of AD patients and enhancing their self-care ability of daily living has generated [26, 27]. It is an appropriate strategy to add some effective compounds derived from plants and animals into the diets so that this fragile population will be able to keep away from the painful side effects which most of drugs have in common. It has been thousands of years since sea cucumbers became a traditional tonic food in China and their medical value has already been acknowledged widely [28]. *A. molpadioides*, material of SC-PC used in this study, is low-value and high yielding with an extensive distribution around the coastal regions of China [29]. Squid (*Sthenoteuthis oualaniensis*) roe, used to extract SQ-PC, is the wastes of processing and so that it is a kind of deep processing to extract phospholipid from this byproduct [30]. Here we investigated three different kinds of phosphatidylcholine (PC) respectively from egg yolk, squid roe and sea cucumber and comparatively studied the effects of different fatty acid composition of PC on brain function. Scopolamine, a traditional anti-cholinergic drugs, is reported to have the capability of impairing spatial learning and memory and influencing the formation of short term memory [31]. In addition, 7 days of SCO-administration has been found to elevate oxidative stress level in rat brain and also increased the neuroinflammatory in the hippocamous [32].

In water maze test, the result of latency showed that egg yolk lecithin (EL), squid PC (SQ-PC) and sea cucumber PC (SC-PC) all can prevent the damage of SCO-administration in mice brain. Otherwise, SQ-PC and SC-PC performed better than EL. Numbers of crossing the platform and time in the target quadrant was used to measure the short term memory of mice. Results revealed that PC improved the decline of memory and in consistent with the place navigation test, SQ-PC and SC-PC with massive n-3 PUFA have more effective functions. According to the behavior test, SQ-PC rich in DHA and SC-PC rich in EPA have a better protection of brain from being damaged in spatial learning and memory by SCO-administration than EL (which barely contained EPA or DHA). Dietary supplement of PC helps slow the aging process and enhance intelligence [33, 34]. As one of the main cerebral material and possessed a high ratio in cerebral fat, it is widely accepted that DHA plays a vital part on maintaining the fluency of cytomembrane and has a relationship with the transmission of nerve signals [34, 35]. The enhanced function of cytomembrane produces amelioration of cell information conducting and leads to raise the activity of brain and nervous system. Recent studied has demonstrated that dietary intake of EPA can be converted into DHA rapidly in brain [35]. EPA and DHA probably adopt some common pathways to prevent cognitive decline. Thus, different fatty composition of PC molecule showed different degree of improvement on behavior test and the species that contained abundant DHA or EPA acted even better.

Ach, a major neurotransmitter in the central cholinergic system, is known to be closely associated with learning and memory. AchE, the hydrolase of Ach, is investigated to evaluate the influence on nerve conduction [36]. There are G1 and G4 isoforms of the enzyme in the brain, both of which were abundant in different brain areas [37]. It has been reported that the G1 isoform of AChE was observed in the brain of AD patients [38]. Several studies suggest that human AChE promotes the fibrilization and deposition of Aβ in pathological aggregates [39, 40]. Decrease of choline acetyltransferase (ChAT) activity, increase of acetylcholin esterase (AchE) activity, low acetyl choline (Ach) content and dysfunction of central nerve system are important pathologies of AD patients [41]. As the result shown, the PC groups displayed significance of reducing the AchE activity in hippocampus, cortex and white matter. Choline, obtained from PC, combines with acetyl-CoA and generates Ach in brain tissues. Supplemental PC in diet could rise the content of choline in brain, and the shrinkage of AchE suppressed the decomposition of Ach, then increased the level of Ach and gained a better effect on cognitive behavior. In addition, it has been reported that n-3 PUFA may have an influence on signal recognition and transduction which was related to the transmission of cholinergic pathways [42]. According to our data, the depression of AchE activity in hippocampus, cortex and white matter was executed better by PC which had high n-3 PUFA content in fatty acids than that with EPA or DHA scarcely included.

Oxidative stress has been one of the primary factors of cognitive decline in neurodegenerative disease for many years. Superoxide anion radicals is the inevitable by-product of the mitochondrial respiration [43]. On the basis of free-radical theory, the reactive oxygen species (ROS) induce the oxidative stress, leading to the damage of protein and DNA, lipid peroxidation and the toxicity against mitochondrial structures. It is so susceptible to oxidation for the long polyunsaturated fatty acid chains of mitochondrial membranes that may lead to the depolarization of membrane and consecutively to mitochondrial impairments [44, 45]. SOD, a momentous antioxidative enzyme, is able to prevent the free radicals from disrupting cells by translating superoxide radical into hydrogen peroxide [46]. As a molecule of cytotoxicity, MDA indicates the degree of membrane lipid peroxidation. The mice with intake of PC acquired better amelioration of oxidative stress (Fig. 3). Interestingly, we found that PC with a mass of DHA was more effective than that with abundant EPA on enhancing the SOD activity in hippocampus and the situation was reverse in cortex. Because of containing EPA and DHA, it was reasonable and turned out that PC with the composition of n-3 PUFA gained more powerful strength of antioxidation than ordinary forms [47]. Nonetheless, there is rarely reported that the distinction of the oxidative function between DHA and EPA is significant in specific tissues and moreover, depending on the result we obtained, the tendency of significance in hippocampus was not as same as that in cortex. There are probably potential mechanisms on the novel phenomenon we can hardly express now. More investigation should be developed to demonstrate the mechanisms. Generally, n-3 PUFA in PC notablely strengthened the anti-oxidative function on the basis of the better performance acted by SQ-PC and SC-PC which were rich in DHA and EPA separately.

MAO, the main enzyme involved in the metabolism of amine such as serotonin, monoamine and catecholamine, has two subunits, MAO-A and MAO-B. Both forms are found with high level in the brain [48]. MAO-B which is the major form in serotonergic neurons and astrocytes has been discovered to closely relate to the impairment of neural functions in central nervous system [49]. The hydrogen peroxide, generated by the reactions of MAO and neural transmitter, could combine with Fe^2+^ and then free radicals were created which promotes the damage of neurone [50]. An increase of MAO activity were detected in the brains of patients with AD [51]. MAO has the ability of increasing the expression of β-secretase and γ-secretase and improving Aβ generation [51] and could be also related to the formation of neurofibrillary tangles [52] when be activated. The alteration of MAO activity in white matter indicated that the supplemental PC had effectively decreased the neural lesions. It is recently reported that 4-weeks’ intragastric administration of fish oil can significantly decline the 5-hydroxyindole acetic acid content and MAO activity and increase serotonin level [53]. In consistent with the present study, SC-PC and SQ-PC, both rich in n-3 PUFA, performed better than EL which mainly had no EPA or DHA on reversing the high MAO activity induced by SCO-administration.

In summary, intraperitoneal injection of scopolamine hydrobromide impaired the brain function seriously in mice and intake of PC could improve the damage effectively. It is widely known that dietary supplement of phospholipid can facilitate the growth and development of infant brain [54]. Intragastric administration of DHA-PC liposome increase the content of unsaturated fatty acid and depressed the TchE activity in mice brain which was accordant in this study [55]. Soybean lecithin and egg yolk lecithin had been reported to improve the PUFA (especially AA and DHA) content and reduce the saturated fatty acid content in rat brain by dietary intake [56]. Boudrault et.al had proved that DHA played a good effect on preventing dementia in four kinds of AD mouse models [57]. Fish oil rich in DHA and EPA was discovered to be capable to decrease anger and anxiety and had been used as hygienical product of brain health for several years [58].

## Conclusions

Different fatty acids composition of PC all could diminish the cognitive decline and biological damage and protect the brain. As PC rich in EPA or DHA showed better improvement than that barely contained, n-3 PUFA partly contributed to the advantageous effects of PC on brain function of demented mice induced by SCO-administration.

## Methods

### Drugs and chemicals

The preparation of Squid (*Sthenoteuthis oualaniensis*, provided by Weihai Boyu Food Company, Shandong Province, China) PC and sea cucumber (*A. molpadioides*, obtained from Nanshan Aquatic Products Market, Shandong Province, China) PC referenced to the Folch Method. Dried powder of squid roes and sea cucumber was extracted with 20-fold volume of the solution of CHCl_3_/MeOH (2:1) for 24 h respectively. Then the resultant filtrate was combined with the 0.88 % (mass fraction) KCl solution into an aqueous condition. The chloroform layer was collected after stratification and used to obtain total lipids by vacuum concentration. Insoluble part of total lipid was dissolved in chloroform at the end of two times of acetone washing. The solution was finally separated on a silica-gel column chromatography to acquire PC component. The purity of PC extracted from squid roe and sea cucumber determined respectively by high performance liquid chromatography (Waters 2695, Milford, MA) were all greater than 95 %. Egg yolk lecithin (EL), obtained from Beijing Aobox Biotechnology co., LTD (Beijing, China), was washed with cold acetone for three times before utilization. According to the fatty acids composition of EL, SQ-PC and SC-PC (Table 1), there was barely DHA and EPA in EL and the content of DHA and EPA were significantly different between SQ-PC and SC-PC. Scopolamine hydrobromide were supplied by Shanghai Harvest Pharmaceutical co., LTD (Shanghai, China). The kits used in the experiments were purchased from Nanjing Jiancheng Bioengineering Institute (Jiangsu Province, China).

**Table 1:** Main fatty acid composition of EL, SQ-PC and SC-PC

FA composition (%)	EL	SQ-PC	SC-PC
Plamitic acid (C16:0)	29.33	27.46	29.28
Plamitoleic acid (C16:1)	1.68	0.54	0.18
Stearic acid (C18:0)	22.2	15.56	14.36
Oleic acid (C18:1)	24.0	1.37	1.64
Lenoleic acid (C18:2)	14.2	--	0.37
EPA (C22:5)	0.32	11.62	38.39
DHA (C22:6)	1.55	35.28	7.35
Other fatty acids	6.75	8.17	8.43

--, none detected, *EPA* eicosapentaenoic acid, *DHA* docosahexaenoic acid

### Animals and diets

Male BALB/c mice (20 ± 2 g, procured from Beijing Vital River Experiment Animal Technology co., LTD.) were housed under temperature (20 ± 2 °C) and relative humidity (50–60 %) with a 12/12 h light/dark cycle (light starting at 8 a.m.).After 1-week adaptation, mice were randomly divided into 5 groups (8 animals per group): model (M) group, control (Con) group, egg yolk lecithin (EL) group, squid PC (SQ-PC) group and sea cucumber PC (SC-PC) group. A modified 93-G Diet was used to feed the mice of Con group and M group. Mice in PC groups were respectively fed with modified 93 M + 0.5 % EL (m/m) diet, modified 93 M + 0.5 % SQ-PC diet and modified 93 M + 0.5 % SC-PC diet. Experimental diets were prepared in accord with recommendations of the American Institute of Nutrition. The ingredients of the experimental diets was displayed in Table 2.

**Table 2:** ingredients of the experimental diets^a^

Ingredients(g kg^−1^)	Con	M	EL	SQ-PC	SC-PC
Casein	200	200	200	200	200
Potato starch	500	500	500	500	500
Sucrose	130	130	130	130	130
Corn oil	70	70	65	65	65
Powdered celluose	50	50	50	50	50
Mineral mix	35	35	35	35	35
Vitamin mix	10	10	10	10	10
Choline bitartrate	2.5	2.5	2.5	2.5	2.5
_DL_-Methionine	3	3	3	3	3
EL	--	--	5	--	--
SQ-PC	--	--	--	5	--
SC-PC	--	--	--	--	5

--, none added, *EL* egg yolk lecithin, *SQ-PC* squid PC, *SC-PC* sea cucumber phosphatidylcholine

^a^prepared according to AIN-93M

### Mice model

**Fig. 5:**
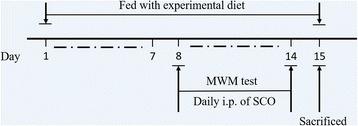
Experemental design and schedule of animal tests. WMW: Morris water maze

The schedule of animal experiments is shown in Fig. 5. The intraperitoneal injection of scopolamine hydrobromide (5 mg/kg) of all mice except Con group initiated on the 8th day of grouped feeding, while Con group mice were injected with the same volume of saline. The injection was daily sustained until the end of test and water maze test started at 1 h after SCO administration.

### Behavior test

Morris Water Maze was made up of a trajectory recording device and a circular pool with a platform. The pool (1.3 m in diameter and 0.5 m in height) was divided into four quadrants and named respectively: I, III, II, IV. With the water (0.3 m in depth) temperature controlled at 21–23 °C, the platform (9 cm in diameter) was submerged 1.5 cm under the water level in the center of quadrant I. A camera linked to a computer was located 2 m above the center of the pool and the movement of the mice trajectory was automatically recorded and analyzed by ANY-maze behavior tracking software (Stoelting Co., Wood Dale, USA). The test included place navigation test and spatial probe test. In place navigation test, each mouse received four trails to be trained to find the platform when being put into the pool respectively from four different quadrants and the sequence was changed every day during the whole training. The time of finding the platform was recorded as latency. If the animal was unable to arrive the hidden platform within 60s test, the latency was recorded as 60s and mouse was placed on the platform for 10s by hand. There was a 20s interval between each trail. The spatial probe test was carried out on the 7^th^ day while the platform was removed and the animals were placed into the pool from quadrant III (opposite to quadrant I) and allowed to search the whole area until the termination. Swim path, time in target quadrant (quadrant I) and numbers of crossing the platform were measured and analyzed.

### Tissues and biological test

One day after the behavior test, mice were sacrificed and their brains were rapidly separated and divided into cortex, hippocampus and white matter. The intact brain tissue was dissected into left and right brain after cerebellum was removed. With the hemisphere being unfolded from the gap of ventricle, the Crescent-shaped hippocampus was drawn out gently. The metathalamus under the gap should be infibulated so that the lamellar white matter attached above the ventricle could be avulsed lightly. The part connected to the white matter was discard to ensure the purity of the cortex.

Brain tissues were respectively extracted with saline at the mass-liquid ratio of 1:9, then the tissue homogenate was centrifuged at 3500 r/min for 10 min. The supernate fluid was applied to measure enzymes activity through the related kits including Acetylcholinesterase (AchE) and superoxide dismutase (SOD) activity in cortex, white matter and hippocampus, monoamine oxidase (MAO) in white matter and malondialdehyde (MDA) content of right brain.

### Statistical analysis

All experiment data were analyzed by SPSS 19.0 and presented as means ± standard errors. Student’s *t* test was used to determine the difference between Con group and M group as well as that between the PC groups and M group, the difference between the PC groups were evaluated by One-way ANOVA with Tukey’s *post hoc* test. Differences were considered significant at *P* < 0.05.

## Abbreviations

Ach: Acetyl choline
AchE: Acetylcholinesterase
ChAT: Choline acetyltransferase
DHA: Docosahexaenoic acid
EPA: Eicosapentaenoic acid
MAO: Monoamine oxidase
MDA: Malondialdehyde
PUFA: Poly unsaturated fatty acids
SCO: Scopolamine
SOD: Super oxide dismutase
